# Synchronous papillary renal neoplasm with reverse polarity and membranous nephropathy: a rare case report

**DOI:** 10.3389/fonc.2025.1512036

**Published:** 2025-05-16

**Authors:** Qingfei Xing, Minglei Zhong, Xiaoxue Li, Tingshuai Cao, Xiaoteng Liu, Fangfei Wang, Li He

**Affiliations:** ^1^ Department of Urology, Central Hospital Affiliated to Shandong First Medical University, Jinan, China; ^2^ Department of Pathology, Central Hospital Affiliated to Shandong First Medical University, Jinan, China; ^3^ Department of Urology, Liaocheng Second People’s Hospital, Liaocheng, China; ^4^ Department of Health, Shandong Province Hospital, Jinan, China

**Keywords:** papillary renal cell carcinoma, reverse polarity, membranous nephropathy, nephrotic syndrome, case report

## Abstract

**Background:**

Papillary renal neoplasm with reverse polarity (PRNRP) is a relatively rare subtype of papillary renal cell carcinoma (PRCC) and is considered to be a tumor of low malignant potential. Membranous nephropathy (MN) is frequently associated with malignant tumors but rarely accompanies renal cell carcinoma. Synchronous papillary renal neoplasm with reverse polarity and membranous nephropathy has not been reported in the current study. (We searched in PubMed, Web of Science databases, Embase, and Medline in the English language from 1970 to October 2024. The keywords used were “papillary renal neoplasm with reverse polarity” and “membranous nephropathy”.)

**Case presentation:**

A 66-year-old man was admitted to the hospital with lower extremity edema and hypertension and presented with nephrotic syndrome including hypoalbuminemia and proteinuria. Enhanced CT scan showed a 3.7*3.0 cm round-like soft tissue density foci at the lower pole of the left kidney, with obvious inhomogeneous enhancement. The patient underwent a laparoscopic partial nephrectomy of the left kidney. Histologic and immunohistochemical results showed typical features of PRNRP, including a papillary structure covered by a single layer of cuboidal cells with finely grained eosinophilic cytoplasm, nuclei that were mostly regular and apically located, and GATA3 (+). The biopsy of pericarcinoma tissue showed membranous nephropathy and glomerular segmental sclerosis. The patient’s nephrotic syndrome resolved and the tumor did not recur or metastasize during 22 months of postoperative follow-up.

**Conclusion:**

We reported a case of synchronous papillary renal neoplasm with reverse polarity with membranous nephropathy. The mechanism of renal tumor-associated nephrotic syndrome is unclear and more medical records are needed for research.

## Background

PRNRP is currently classified as a subtype of PRCC. In 2008 Kunju et al. first described PRCC lined by oncocytic cells with peripheralized low-grade nuclei (1). In 2019, Al-Obaidy et al. proposed the new term “ papillary renal neoplasm with reverse polarity “ and subsequent studies identified unique features including papillary structures covered by a single layer of cuboidal cells, granular eosinophilic cytoplasm, nuclei that were mostly regular and apically situated with inconspicuous nucleoli, GATA3 and L1CAM positivity, negative Vimentin staining, frequent KRAS gene mutations (2, 3). Most patients have a good prognosis. Membranous nephropathy was a glomerular disease characterized by thickening of glomerular capillary walls due to immune complex deposition. Patients lost a large amount of protein in the urine and developed nephrotic syndromes such as decreased serum albumin levels and generalized edema (4). Previous studies have shown that some malignant tumors such as gastrointestinal tumors and lung cancer can cause glomerular disease (5, 6). However, there were few reports of nephrotic syndromes caused by renal carcinoma, and the mechanism between renal carcinoma and glomerular disease was unclear. Here we describe for the first time a case of synchronous PRNRP with membranous nephropathy.

## Case presentation

**Figure 1:**
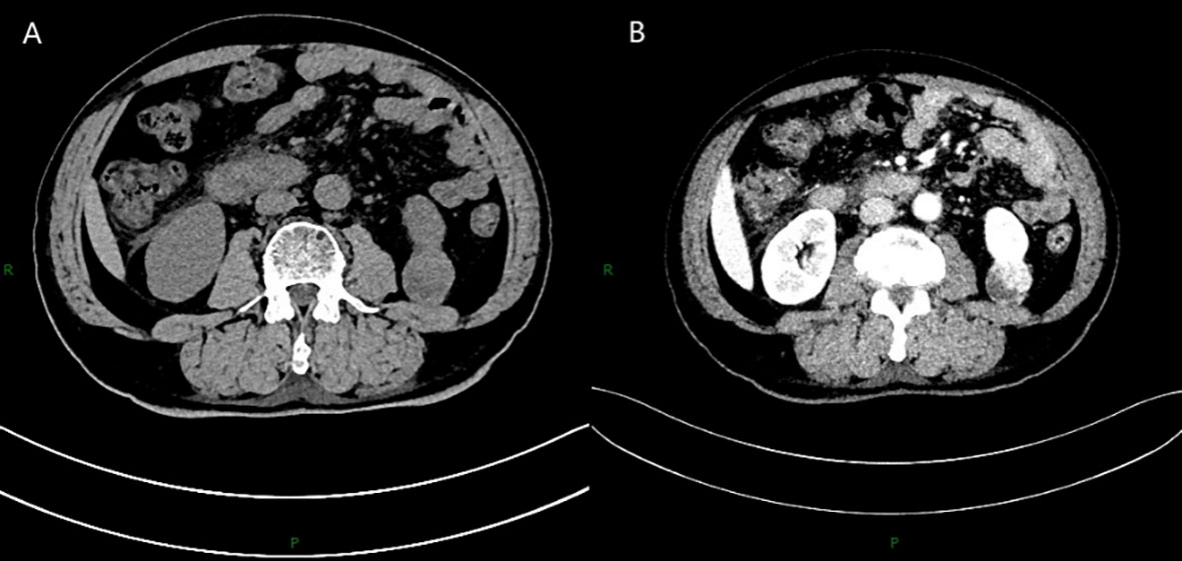
CT plain scan showed a 3.7*3.0 cm round soft tissue density focus at the lower pole of the left kidney **(A)**, and the enhancement scan showed the lesion was inhomogeneous enhancement **(B)**.

A 66-year-old man was hospitalized with symptoms of lower extremity edema and hypertension, and ultrasonography revealed a solid mass in the left kidney. CT plain scan showed a 3.7*3.0 cm round soft tissue density focus at the lower pole of the left kidney (**Figure 1A**), and the enhancement scan showed the lesion was inhomogeneous enhancement (**Figure 1B**). Laboratory tests showed serum albumin 23.1 g/L, 24-hour urine protein quantification 3.018 g (reference value <0.140), AFP 7.8 ng/mL, CEA 5.15 ng/mL, NSE 22.62 ng/mL. Tests for PLA2R serology and other autoimmune markers were negative. The patient underwent a laparoscopic partial nephrectomy.

**Figure 2:**
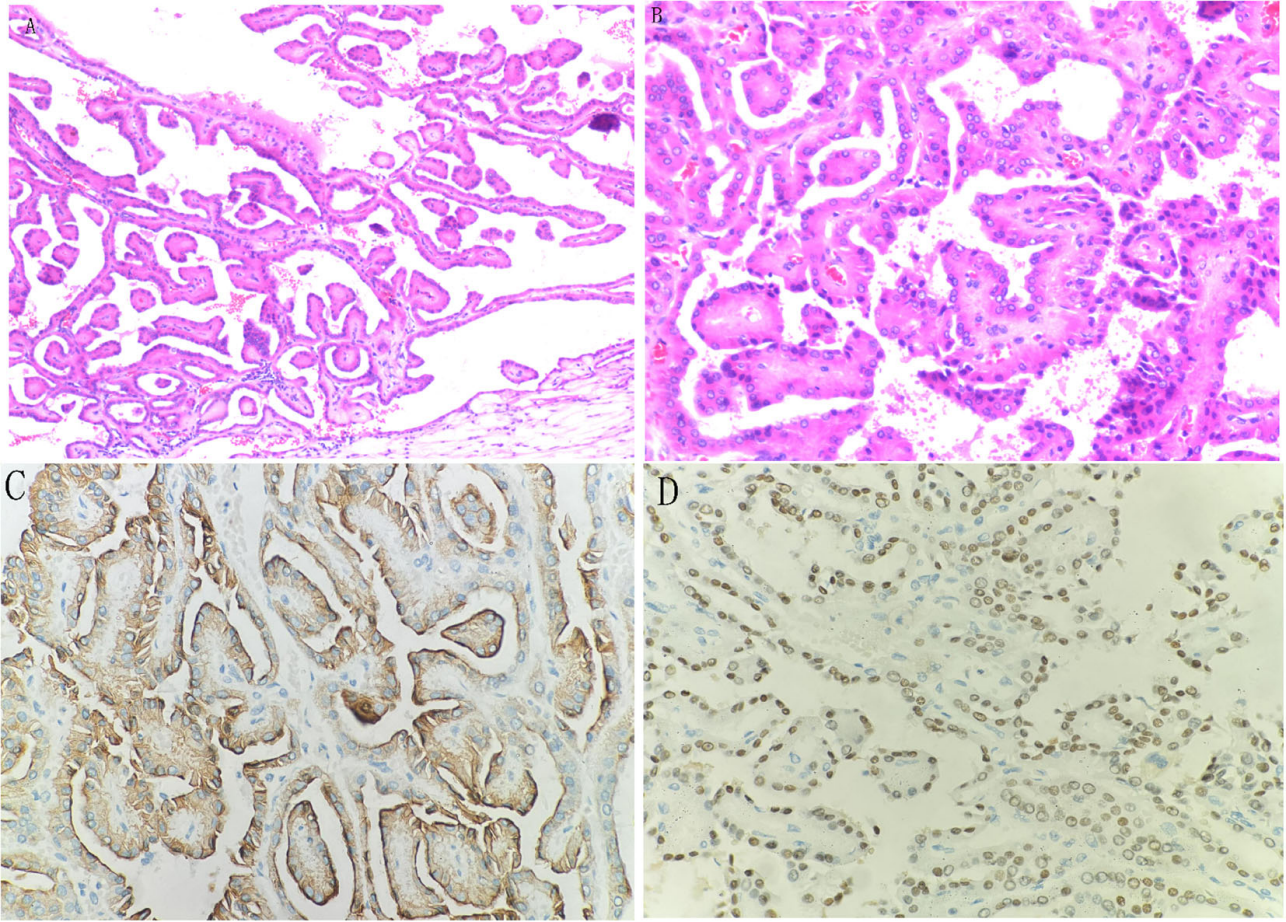
Histologically, the tumors were accompanied by a fine fibrovascular core of branching papillae **(A)**, and most tumor cells were arranged in a monolayer on the papillary basement membrane. Most of the cells were cuboidal, with fine-grained eosinophilic cytoplasm. The nuclei were located on the apical surface of the cells, mostly small and non-overlapping, with regular nuclear contours and inconspicuous nucleoli **(B)**. Tumors tissue immunohistochemistry showed CK7 (+) **(C)**, GATA3 (+) **(D)**.

Macroscopically, the tumor was dissected with clear boundaries, and a cystic solid mass measuring about 3.5*3.5 cm with gray-red and gray-yellow papillary protrusions in the solid area was seen on the cut surface. Histologically, the tumors were accompanied by a fine fibrovascular core of branching papillae (**Figure 2A**), and most tumor cells were arranged in a monolayer on the papillary basement membrane. Most of the cells were cuboidal, with fine-grained eosinophilic cytoplasm. The nuclei were located on the apical surface of the cells, mostly small and non-overlapping, with regular nuclear contours and inconspicuous nucleoli (**Figure 2B**). Tumors tissue immunohistochemistry showed CK7 (+) (**Figure 2C**), EMA (+), GATA3 (+) (**Figure 2D**), PAX-8 (+), CD10 (-), CA9 (-), Vimentin (-), CD117 (-), TFE-3 (-), WT-1 (-), weak positivity for P504S and P53, and 5% Ki-67.

**Figure 3:**
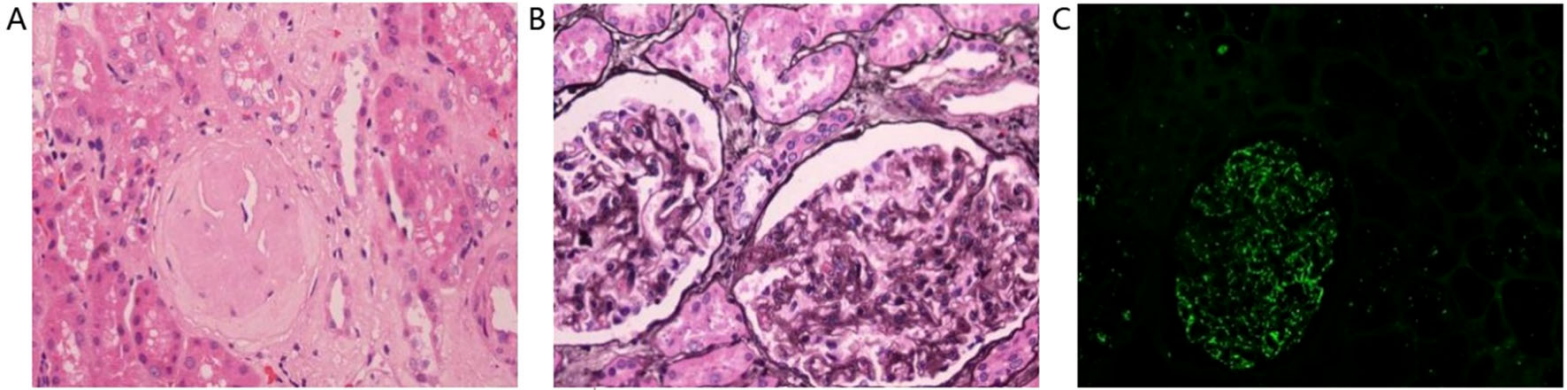
Histopathological analysis of the renal tissue surrounding the tumor showed glomerulosclerosis in 47 out of 284 glomeruli and segmental sclerosis in 10 glomeruli **(A)**. PASM staining showed mild hyperplasia of glomerular mesangial cells and stroma, thickening of the basement membrane, numerous spikelike structures and chain-like structures, and the deposition of subepithelial eosinophilic deposit **(B)**. Immunofluorescence results showed fine granular deposits of IgG, IgM, and C1q along capillary loops. IgA, C3(-), and C3 in the walls of small arterioles were positive **(C)**.

Histopathological analysis of the renal tissue surrounding the tumor showed glomerulosclerosis in 47 out of 284 glomeruli and segmental sclerosis in 10 glomeruli (**Figure 3A**). PASM staining showed mild hyperplasia of glomerular mesangial cells and stroma, thickening of the basement membrane, numerous spikelike structures and chain-like structures, and the deposition of subepithelial eosinophilic deposit (**Figure 3B**). Immunofluorescence results showed fine granular deposits of IgG, IgM, and C1q along capillary loops. IgA, C3(-), and C3 in the walls of small arterioles were positive (**Figure 3C**).

The patient was followed up for 22 months after tumor resection with no recurrence or metastasis of the tumor and normal renal function.

## Discussion

PRCC is the second most common renal cell carcinoma and has traditionally been classified into type 1 and type 2 based on histological differences (7). However, in actual clinical practice, a large proportion of tumors, including PRNRP, exhibited overlapping features (8). Studies have shown that PRNRP accounts for approximately 4% of the total number of previously diagnosed PRCCs (9). PRNRP was often considered to have indolent biological behavior due to its papillary growth pattern, low-grade nuclei, fewer chromosomal abnormalities, and good survival outcomes. The study by Wang et al. reported firstly one case of recurrent PRNRP at 6 months postoperatively, and no cases of metastasis and death during the follow-up period (10). Some reported cases of PRNRP showed histologic features of localized invasion of peripheral nerves and renal parenchyma (11).Therefore, the biological behavior of PRNRP needs to be further investigated.

The typical pathological features of membranous nephropathy (MN): abnormal basement membrane, immunofluorescent IgG with or without C3 along the basement membrane deposition, subepithelial electron dense deposition.25% to 30% membrane Nephropathy is secondary to malignant tumors (solid tumors such as lung cancer, bowel cancer, breast cancer), infections (hepatitis B, malaria, etc.), connective tissue diseases (systemic lupus erythematosus, etc.) and drugs/poisons (NSAIDs, Chinese medicines, heavy gold genus etc.).Membranous nephropathy with no secondary factors is called idiopathic membranous nephropathy idiopathic nephropathy (IMN) and also known as primary membranous renal disease nephropathy(PMN).

We reported a patient with PRNRP combined with membranous nephropathy(MN). Previous studies have shown that MN is often associated with malignancy, and the prevalence of cancer in patients with MN has been estimated to be 10% (12). MN often occurred in combination with lung cancer, gastrointestinal tract tumors, and prostate cancer, but less with renal cancer (13). It is difficult to confirm whether MN is primary or secondary in clinical practice, but we can attempt to make inferences based on the available evidence. In our case, the patient’s symptoms of proteinuria improved significantly after surgery. A similar case report described a patient with MN and renal cancer, where MN was cured by resection of the primary tumor (14). On the other hand, tumor development is a relatively slow process, but our patient presented with symptoms of proteinuria and edema for only 2 days, which suggested a late onset of nephrotic syndrome. The above evidence supported to some extent the conclusion that MN is secondary to PRNRP.

The mechanism of tumor-associated nephropathy has not been elucidated. There was experimental evidence that proteinuria could occur in loaded tumor-bearing animals, with diffuse deposition of IgG in the glomeruli and loss of podocyte peduncles (15). The prevailing view was that it was the result of the deposition of antibody complexes to tumor-associated antigens in the glomeruli. It has also been suggested that RCC patients are more susceptible to immune complex damage caused by exogenous or endogenous antigens secondary to altered immune function (16). Our report indicated a relationship between tumors and MN, and further studies are needed to elucidate the mechanism of this association.

The diagnosis of MN usually precedes the diagnosis of the associated cancer, and some clues may suggest the presence of cancer in patients with membranous nephropathy, such as negative anti-PLA2R1 antibodies, prevalent IgG1 and IgG2 deposits, or more than 3 inflammatory cells per glomerulus on renal biopsy (17). With the discovery of anti-phospholipase A2 receptor (PLA2R) antibodies and anti-platelet domain protein type I 7A (THSD7A) antibodies, the study and diagnosis of PMN entered the molecular era (18, 19).The understanding of membranous nephropathy has developed from pathological morphology to molecular level. Serum anti-PLA2R antibody has high specificity in the diagnosis of membranous nephropathy. There is still disagreement on whether serum anti-PLA2R and THSD7A antibody detection can replace kidney biopsy. At the 2019 KDIGO meeting, it was proposed that risk stratification should be performed first for patients with positive anti-PLA2R antibodies, and low-risk patients could not temporarily undergo kidney biopsy and be given supportive treatment first. Renal biopsy is recommended for patients who are at high risk or who are reassessed as high risk during follow-up (20). PLA2R positivity is common in PMN but is not a specific marker of PMN.

At present, the treatment of membrane nephropathy is becoming more and more standardized, and the KDIGO guidelines recommend that the timing and regimen of immunosuppressive therapy be determined according to the risk of kidney disease progression and the risk of complications of immunosuppressive use. For renal function progression, it is possible to benefit from starting immunosuppressive therapy as early as possible in patients with deterioration and no contraindication of immunosuppressive therapy. For patients with mild symptoms and stable renal function, immunosuppressive therapy should be delayed as long as possible (20).For immunosuppressive regimens, hormone combined alkylating agent regimens, rituximab regimens, and Calcineurin inhibitor (CNI) regimens were all first-line regimens. Currently, only hormonal combined alkylating agent regimens have evidence to delay entry to End-stage renal disease (ESRD) (21).

CNI regimens and rituximab regimens for membranous nephropathy evidence of therapeutic effect is limited to induced remission of nephrotic syndrome (21).

Therapeutically, patients with renal tumors combined with MN need to decide on the order of treatment on an individual basis. Treatment of MN may contribute to more rapid progression of preexisting tumors. For example, glucocorticoids can interfere with inflammation, inhibit antigen presentation, and suppress cellular and humoral immunity (18). On the other hand, in patients with renal insufficiency, surgical complications may further impair their renal function. The reason we chose to deal with the renal tumor first in our case was that the patient’s preoperative renal function was essentially normal and the PRNRP was small. Therefore, the patient was suitable for nephron-sparing surgery and the follow-up results showed no deterioration of renal function after surgery. The patient was not treated for MN after surgery, and the postoperative review showed that the symptoms of MN improved. This requires further research and exploration.

## Conclusions

In conclusion, we reported a case of synchronous papillary renal neoplasm with reverse polarity with membranous nephropathy. The mechanism of renal tumor-associated nephrotic syndrome is unclear. PRNRP were mostly stage T1 tumors with good prognosis and were more suitable for nephron-sparing surgery considering the damage of MN to renal function.

## Data Availability

The datasets presented in this study can be found in online repositories. The names of the repository/repositories and accession number(s) can be found in the article/supplementary material.
